# Stereotactic central/core ablative radiation therapy: results of a phase I study of a novel strategy to treat bulky tumor

**DOI:** 10.3389/fonc.2024.1364627

**Published:** 2024-05-24

**Authors:** Jun Yang, Qiuxia Lu, Weihua Qi, Ryann D. Kolb, Lei Wang, Yuan Li, Sida Li, Yihui Lin, Jiayi Liu, Waleed Mourad, Farzaneh MirkhaghaniHaghighi, Tubin Slavisa, Xiaodong Wu, Wei-Ciang You, Eddy Yang, Alex Hanlon, Alan Zhu, Weisi Yan

**Affiliations:** ^1^ Departmentof Radiation Oncology, Junxin Oncology Group, Foshan, China; ^2^ Department of Radiation Oncology, Foshan Chancheng Central Hospital, Foshan, China; ^3^ Department of Statistics, Virginia Tech, Blacksburg, VA, United States; ^4^ Department of Radiation Oncology, Quanzhou Binhai Hospital, Quanzhou, China; ^5^ Taichung Veterans General Hospital, Department of Radiation Oncology, Taichung, Taiwan; ^6^ Department of Radiation Oncology, University of Pennsylvania, Philadelphia, PA, United States; ^7^ Department of Radiation Medicine, Markey Cancer Center – UK Chandler Medical Center, Lexington, KY, United States; ^8^ College of Medicine, University of Kentucky, Lexington, KY, United States; ^9^ Cameroon Oncology Center, Douala, Cameroon; ^10^ Medaustron Center for Ion Therapy, Wiener Neustadt, Austria; ^11^ Department of Radiation Oncology and Radiation Therapy, Heidelberg University Hospital, Heidelberg, Germany; ^12^ Department of Radiation Oncology, Executive Medical Physics Associates, Miami, FL, United States; ^13^ Mayo Clinic Alix School of Medicine, Scottsdale, AZ, United States

**Keywords:** ROB-D-23-00930 clinical investigation -Other SFRT, SCART, SBRT, phase 1, bulky tumor

## Abstract

**Purpose:**

Bulky tumor remains as a challenge to surgery, chemotherapy and conventional radiation therapy. Hence, in efforts to overcome this challenge, we designed a novel therapeutic paradigm via strategy of Stereotactic Central/Core Ablative Radiation Therapy (SCART).), which is based on the principles of SBRT (stereotactic body radiation therapy and spatially fractionated radiation therapy (SFRT). We intend to safely deliver an ablative dose to the core of the tumor and with a low dose at tumor edge. The purpose of the phase 1 study was to determine dose-limiting toxicities (DLT)s and the Maximum Tolerated Dose (MTD) of SCART.

**Methods and materials:**

We defined a SCART-plan volume inside the tumor, which is proportional to the dimension of tumor. VMAT/Cyberknife technique was adopted. In the current clinical trial; Patients with biopsy proven recurrent or metastatic bulky cancers were enrolled. The five dose levels were 15 Gy X1, 15Gy X3, 18GyX3, 21GyX3 and 24GyX3, while keeping the whole tumor GTV’s border dose at 5Gy each fraction. There was no restriction on concurrent systemic chemotherapy agents.

**Results:**

21 patients were enrolled and underwent SCART. All 21 patients have eligible data for study follow-up. Radiotherapy was well tolerated with all treatment completed as scheduled. The dose was escalated for two patients to 24GyX3. No grade 3 or higher toxicity was observed in any of the enrolled patients. The average age of patients was 66 years (range: 14**–**85) and 13 (62%) patients were male. The median SCART dose was 18Gy (range: 15 - 24). Six out of the 18 patients with data for overall survival (OS) died, and the median time to death was 16.3 months (range: 1 - 25.6). The mean percent change for tumor shrinkage between first visit volumes and post-SCART volumes was 49.5% (SD: 40.89, p-value:0.009).

**Conclusion:**

SCART was safely escalated to 24 GyX 3 fractions, which is the maximum Tolerated Dose (MTD) for SCART. This regimen will be used in future phase II trials.

## Introduction

Patients afflicted with bulky tumors often face a grim prognosis, typically necessitating palliative treatments. Conventional therapeutic modalities such as surgery, traditional chemotherapy, and standard radiotherapy offer limited efficacy in addressing the oncologic challenges posed by these sizable tumors. Nonetheless, enhancing local disease control in bulky tumors can markedly enhance both overall survival (OS) and quality of life (QOL) (1, 2). Stereotactic body radiotherapy (SBRT) presents a promising avenue for achieving superior local control owing to its ability to administer a high biological equivalent dose (BED) (3). However, a critical issue arises with the treatment of bulky disease, characterized by its aggressive biological behavior necessitating escalated radiation doses. The substantial tumor size poses a formidable obstacle to the utilization of SBRT, thereby compromising local control and response rates due to the heightened risk of collateral damage to surrounding tissue (4, 5).

SBRT demonstrates notable efficacy in tumor control, particularly in targeting small lesions, owing to its ablative radiation dose nature, which challenges conventional linear-quadratic (LQ) models (6). This efficacy extends beyond mere DNA double-strand damage, as elucidated by the LQ model, to encompass additional biological effects such as immune modulation, vascular disruption, and the elusive abscopal effect (7–9). We see radiation as a medicine that can modulate biological systems in different manners when applied creatively in different dose, target, fractionation and timing. Numerous studies have underscored the potential of harnessing the potent immune-modulatory properties of ablative radiotherapy in conjunction with immuno-regulatory agents as a practical strategy for augmenting the efficacy of radiotherapy and improving cancer outcomes (10–13).

Spatially Fractionated Radiation Therapy (SFRT) is a novel approach that builds upon the principles of Stereotactic Body Radiation Therapy (SBRT) (14). SFRT introduces a significant conceptual shift by administering radiation doses in a non-uniform manner across whole or partial tumor volumes, thereby offering a platform for enhanced creativity and supplementary applications to conventional radiation therapy protocols. Variants of SFRT, such as GRID therapy (15, 16) and Lattice Therapy (17, 18), have proved potential advantages in the management of bulky tumors, despite the need for further investigation into best dosing and fractionation strategies. However, a notable challenge lies in the generation of multiple “hot spots” to encompass the entirety of bulky tumors, which may need limiting the size of individual hot spots. Consequently, the total volume covered by these hot spots (in the form of strips or islands) remains comparatively small compared to the overall volume of the bulky tumors.

The studies conducted by Tubin et al. (19) and Luke et al. (20) have made noteworthy contributions to the exploration of partial-volume Stereotactic Body Radiation Therapy (SBRT), particularly within the context of reirradiation. Consequently, it is imperative to find the novelty of the proposed SCART technique compared to these existing methodologies. Tubin et al. employ biological volumes, which may pose challenges in resource-constrained settings, and standardization of dose planning still is elusive. Conversely, Luke et al. use a dose range of 8–12Gy, but doses>10Gy typically induces vascular damage to tumors, thus dose escalation intratumorally is still yet to be explored (21).

Our aim is to optimize the intra-tumoral high-dose area to achieve maximal ablative effects in a manner that is both safe and systematically reproducible for planning and execution, thereby distinguishing our approach from previously published techniques.

## Concept of SCART

Considering the promising clinical outcomes observed with Spatially Fractionated Radiation Therapy (SFRT), including response rates exceeding 90% and a complete response (CR) rate of 27% when administered as a standalone treatment (15), targeting a partial tumor with SFRT may confer significant benefits. Furthermore, the induction of abscopal and bystander effects has been documented when employing SBRT to target the hypoxic core of tumors with elevated levels of radiation (19).

We postulate that increasing the proportion of the tumor’s central core receiving ablative doses heightens the likelihood of triggering biological effects. Concurrently, combining low-dose fractionated radiation to the tumor periphery may mitigate some of the immunosuppressive effects associated with high-dose radiation to the tumor edge.

Our aim is to optimize the high-dose region within the tumor core to achieve enhanced ablative effects, particularly within regions housing cancer stem cells or highly resistant progenitors. Additionally, we seek to democratize access to this method by making it available to centers lacking routine access to PET-based planning, thereby standardizing the planning process through an equation-based approach rooted in tumor size and shape. This standardized methodology ensures repeatability and reproducibility across treatment sessions.

Hence, we introduce a novel treatment approach termed Stereotactic Centralized/Core Ablative Radiation Therapy (SCART) for managing bulky or metastatic tumors, which is founded upon the principles of Spatially Fractionated Radiation Therapy (SFRT). SCART entails employing Stereotactic Body Radiation Therapy (SBRT) techniques to administer an ablative radiation dose to a sizable central segment of the target, designated as the SCART-PTV, while concurrently minimizing radiation exposure to surrounding healthy tissue. Emphasizing tissue safety as our foremost priority, our objective is to capitalize on modern technological advancements to expand the proportion of the target volume amenable to high-dose radiation delivery.

We also performed a prospective, multi-center, dose-escalation phase 1 trial of SCART with and without additional External Beam Radiation Therapy (EBRT) for bulky metastatic or recurrent cancer. The study’s purpose was to determine the safety, dose-limiting toxicities (DLT)s and the Maximum Tolerated Dose (MTD) of SCART.

## Phase I clinical trial

This protocol was approved by Foshan chancheng hospital as a phase 1 trial in 5/27/2020 and by IRB of QuanZhou BinHai Hospital, Fujian, China. It was also registered as phase 1 trial in clinical trial.org in 2021.

## Eligibility

Patients must be able to understand and sign a written informed consent document. Adult patients aged 18 years or older presenting with recurrent or metastatic bulky cancers, confirmed histologically or cytologically as nonhematological in nature, were eligible for enrollment in this study. Surgical ineligibility or patient refusal to undergo surgery constituted inclusion criteria. There were no restrictions on prior chemotherapy or radiation therapy. Concurrent use of chemotherapy or immunotherapy with SCART was not restricted, as many patients had exhausted alternative treatment modalities prior to enrollment, and the study did not aim to investigate combination effects of SCART with systemic therapy.

Patients were prospectively enrolled in an Institutional Review Board-approved clinical trial following confirmation of cancer diagnosis via biopsy. Inclusion criteria included measurable disease documented by CT and/or PET imaging, with lesions possessing a shortest axis of 3 cm or greater amenable to SCART radiation. We employed a cutoff criterion of greater than 5 cm in any axis for defining bulky disease in extracranial lesions, whereas a cutoff of 3 cm was applied for intracranial lesions.

All hematologic tumors were excluded from the study. Additionally, patients had to be at least 18 years old with a life expectancy of at least 6 months to ensure evaluability of study endpoints. A Zubrod/GOG performance status of 0 or 1 was required for enrollment, along with normal organ and marrow function as defined by specified laboratory parameters.

Laboratory evaluations, including complete blood count with differential, liver function tests, and serum creatinine, were conducted to assess eligibility. Exclusion criteria comprised pregnancy or breastfeeding, comorbidities associated with a life expectancy of ≤ 6 months, or uncontrolled intercurrent illnesses such as active infection, symptomatic congestive heart failure, unstable angina pectoris, cardiac arrhythmia, or psychiatric illness/social situations impeding compliance with study requirements. Patients with active Crohn’s disease or inflammatory bowel disease (IBD) were also excluded.

## Radiation therapy

Stereotactic Core Ablative Radiation Therapy (SCART) was achievable through the linear accelerator (Linac) and robotic radiosurgery system (Cyberknife). Among these, Linac-based Volumetric Modulated Arc Therapy (VMAT) stands out as the most utilized approach in SCART applications.

Typically, SCART is prescribed at doses ranging from 15 to 24 Gy over three fractions every other day, with constraints on the dose to surrounding tissues set at 3 Gy or 5 Gy per fraction. Most patient finished in 1–2 weeks with SCART therapy, several patients came back after prolonged time for additional SCART treatment.

### Definition of GTV and SCART-PTV

**Figure 1:**
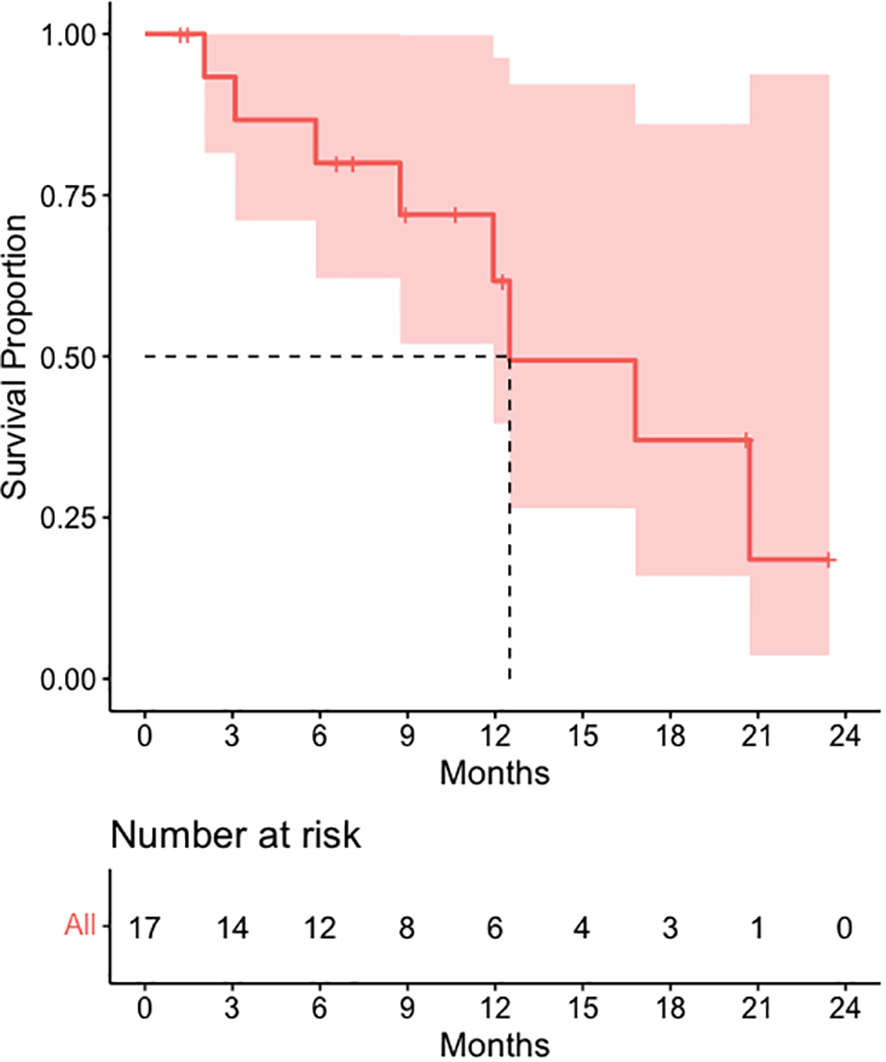
Kaplan-Meier Curve for Overall Survival (N= 17 patients).

Patients underwent CT based simulation with 2 mm thin slice CT for treatment planning. Contrasted CT, MRI and PET/CT are often acquired and fused with sim CT to help tumor and organ at risk (OAR) target delineation and contouring. Gross target volume (GTV) is defined as bulky visible disease in these images. Clinical target volume (CTV) is the same as GTV and not highlighted in this study. SCART-PTV or SCART is defined as the region inside of GTV and receives ablative dose. (**Figure 1**) In the SCART planning, SCART-PTV, but not the whole GTV nor TTV, is the true target of ablation and is pre-defined at the core of GTV prior to the planning optimization. In our design as **Figure 1**, the maximum opening of radiation arc is limited to the size of SCART-PTV. These radiation fields only converge at SCART-PTV, but not at TTV, and this arrangement will generate the highest possible dose gradient in TTV, the region between SCART-PTV and GTV border.

### Treatment planning

Five dose-levels were proposed, with starting dose level 1 of 15 Gy to primary central spot PTV in one fraction, level 2 of 15Gy X3, level 3 of 18GyX3, Level 4 of 21GyX3, Level 5 of 24GyX3, while keeping the whole tumor GTV’s border dose at 5Gy each fraction. An additional two 5Gy per fraction SBRT were added to certain patients, so that the total GTV border dose is 5Gyx5, deemed as a safe dose to most of the surrounding tissue. If SBRT dose is not added, these patients would receive another course of SCART in 6 weeks. Heterogeneity corrections were used in the definitions of all doses. Non-anatomical dose constraint structures were incorporated to aid the optimization process in minimizing the dose to critical structures. One or two co-planar 360 degree 6 MV photon arcs were used with VMAT technology. The maximum field size was limited to SCART-PTV in the optimization, so that all the radiation fields from 360 degrees only irradiate and intersect at SCART-PTV, but not the whole GTV. This will generate the maximum dose gradient between SCART-PTV and GTV border. The SCART-PTV’s shape is typically a spindle shape, and its dimension is proportional to the GTV dimension. The proportion is determined by the prescription dose (15~24Gy) and border dose (5Gy). The higher the prescription dose is, the smaller the proportion is. Many tests were done, and the recommended proportion is listed in **Table 1**.

**Table 1:** Dose escalation scheme for each cohort, three patients are initially enrolled into a given dose cohort. If there is no dose-limiting toxicity (DLT) seen in any of these participants, the trial can proceed to enroll additional participants into the next higher dose cohort.

Escalation level	RX of SCART	Constraints Dose at GTV border	*SCART’s Diameter GT ‘s Diameter*	% of GTV Vol
**-1**	15 Gy x1	5 Gy x 1	36%	10.6%
**1**	15 Gy x2	5 Gy x 2	36%	10.6%
**2**	15 Gy x3	5 Gy x 3	36%	10.6%
**3**	18 Gy x3	5 Gy x 3	27%	6.3%
**4**	21 Gy x3	5 Gy x 3	24%	4.5%

### Delivery

High-dose SCART radiation therapy was delivered on day 1, then SCART was delivered every other day until the intended dose level was achieved or discontinuation due to toxicity. The radiation dose specifications are provided in **Supplementary Material 2**. Cone beam (CBCT) was acquired before every treatment.

## Concurrent medication and supportive care

There were no restrictions imposed on the concurrent use of prescription medications during SCART treatment. Patients could receive chemotherapy and/or immunotherapy concurrently with SCART. Additionally, any appropriate supportive care medication or treatment was allowed.

## Follow-up

Following the completion of radiation treatment, patients underwent a follow-up evaluation 6 weeks later, followed by subsequent assessments every 2 months for at least 6 months. More frequent follow-up visits were conducted if clinically indicated, with subsequent visits scheduled every 2 to 4 months at the discretion of treating physicians. Tumor response was evaluated using CT, MRI, or PET scans, and assessments were performed every 2 to 3 months utilizing Response Evaluation Criteria in Solid Tumors 1.1 (RECIST) principles. The timing of recurrence was determined based on the first observation of progressive abnormalities on imaging.

## Radiation toxicity analysis

The primary objective of the study was to ascertain dose-limiting toxicities (DLTs) and establish the Maximum Tolerated Dose (MTD) of SCART. DLTs were defined as grade 3 or higher non-hematologic adverse events attributable to radiation treatment, graded according to Common Terminology Criteria for Adverse Events version 4.0 (CTCAE v.4.0), occurring within 90 days following treatment initiation. The MTD was defined as the highest dose level at which no more than 1 of 6 patients or 0 of 3 patients experienced DLT within 90 days of follow-up. This escalation rule adhered to the traditional 3 + 3 design, where a DLT rate of less than 33% at the current dose level warranted further dose escalation.

## Statistical methods

Descriptive statistics were employed to characterize demographic variables, including age and sex, as well as tumor characteristics such as tumor site, histology, stage, and treated site. Subsequently, patients’ treatment histories, encompassing indicators for systemic therapy, chemotherapy, and immunotherapy, were detailed in tables along with indicators for key outcomes such as Overall Survival (OS), Progression-Free Survival (PFS), and Local Recurrence (LR), as well as the corresponding outcome measures themselves [OS, time to LR, and Time to Surgery (TS)] (**Tables 2A**, **2B**).

**Table 2A:** Demographics and treatment characteristics (N=17 patients).

Patient Level Characteristics	(N= 17 patients)
Demographics	
**Age, mean (SD)**	65.65 (17.39)
**Male Sex, n (%)**	10 (58.8)
Treatment Characteristics	
**Systemic therapy = Yes, n (%)**	9 (56.3)
**Chemotherapy = Yes (%)**	2 (11.8)
**Immunotherapy = Yes (%)**	6 (35.3)
**Died, n (%)**	8 (47.1)
**Overall Survival (months) (median [IQR])**	8.94 [5.85, 12.49]

**Table 2B:** Tumor characteristics (N=20 tumors among 17 patients).

Tumor Level Characteristics	(N= 20 tumors)	
Treatment Characteristics		
Tumor Site, n (%)		
** Breast**	2 (10.0)	
** Chest wall**	1 (5.0)	
** Endometrium**	1 (5.0)	
** Gallbladder**	1 (5.0)	
** Liver**	5 (25.0)	
** Lung**	5 (25.0)	
** Mediastinum**	1 (5.0)	
** Pancreas**	1 (5.0)	
** Right Temporal Lobe**	1 (5.0)	
** Ureter**	1 (5.0)	
** Ventricle**	1 (5.0)	
Tumor Histology, n (%) (N= 17 tumors*)		
** Adenocarcinoma**	4 (23.5)	
** Epithelial**	1 (5.9)
** Fibroma**	1 (5.9)	
** Hepatocarcinoma**	1 (5.9)	
** Hepatocellular Carcinoma**	3 (17.6)	
** Invasive Ductal Carcinoma**	2 (11.8)	
** Leiomyosarcoma**	1 (5.9)	
** Malignant Mesenchymoma**	1 (5.9)	
** Mesothelioma**	1 (5.9)	
** Squamous cell carcinoma**	2 (11.8)	
Tumor Stage, n (%)		
** II**	1 (5.0)	
** III**	3 (15.0)	
** IV**	14 (70.0)	
** Tis**	2 (10.0)	
Treated Site, n (%)		
** 4th ventricle**	1 (5.0)	
** Brain 1**	1 (5.0)	
** Brain 2**	1 (5.0)	
** Intra-abdomen**	1 (5.0)	
** Liver**	5 (25.0)	
** Lung**	4 (20.0)	
** Mediastinum**	1 (5.0)	
** Pancreas**	2 (10.0)	
** Pelvis**	1 (5.0)	
** Right breast**	2 (10.0)	
** Right chest wall**	1 (5.0)	
** GTV volume (cm^3^), median [IQR]**	166.45 [74.75, 300.58]	
SCART Dose (Gy), n (%)		
** 15**	10 (50.0)	
** 18**	5 (25.0)	
** 21**	3 (15.0)	
** 24**	2 (10.0)	
SCART Dose at tumor edge (% of total dose), n (%)		
** 20**	4 (20.0)	
** 30**	15 (75.0)	
** 40**	1 (5.0)	
LR, n (%) (N= 18 tumors**)	1 (5.6)	

*3 tumors were removed due to missing data for tumor histology **2 tumors were removed due to missing data for local recurrence.

Continuous variables such as age and Gross Target Volume (GTV) size were summarized using means and standard deviations. Categorical variables, including sex, tumor site, stage, treated site, systemic therapy, chemotherapy, immunotherapy, and indicators for death, PFS, and LR, were presented using frequencies and percentages. Medians and ranges were utilized for describing skewed variables such as SCART dose, SCART dose at tumor edge, and outcome variables like OS and Time to LR.

OS, PFS, and time to LR were estimated employing Kaplan-Meier methodology, and their distributions within the overall sample were visually depicted using Kaplan-Meier curves. A one-sample t-test was conducted to compare tumor volumes pre- and post-SCART, with visual aids including a boxplot and spaghetti plot facilitating this comparison.

## Outcome analysis

The outcomes of interest were overall survival, time to local recurrence, and changes in tumor volume from pre- to post-SCART treatment.

### Overall survival

OS was measured in months from the time of study enrollment, also known as the patient’s first SCART date, to date of death or last follow-up.

### Time to local recurrence

LR was measured in months from the time of study enrollment, also known as the patient’s first SCART date, to date of local recurrence or last follow-up.

### Tumor change

TC was measured by the percent change in tumor volume from the volume (cm^3^) at first visit to the final tumor volume (cm^3^) post SCART.

Tumor Shrinkage (TS): TS was measured by the percent change in tumor volume from the volume (cm^3^) at first visit to the final tumor volume (cm^3^) post SCART were 7 patients included in this analysis due to missing SCART dates and one patient (JM) who was an outlier.

## Statistical methods

**Figure 2:**
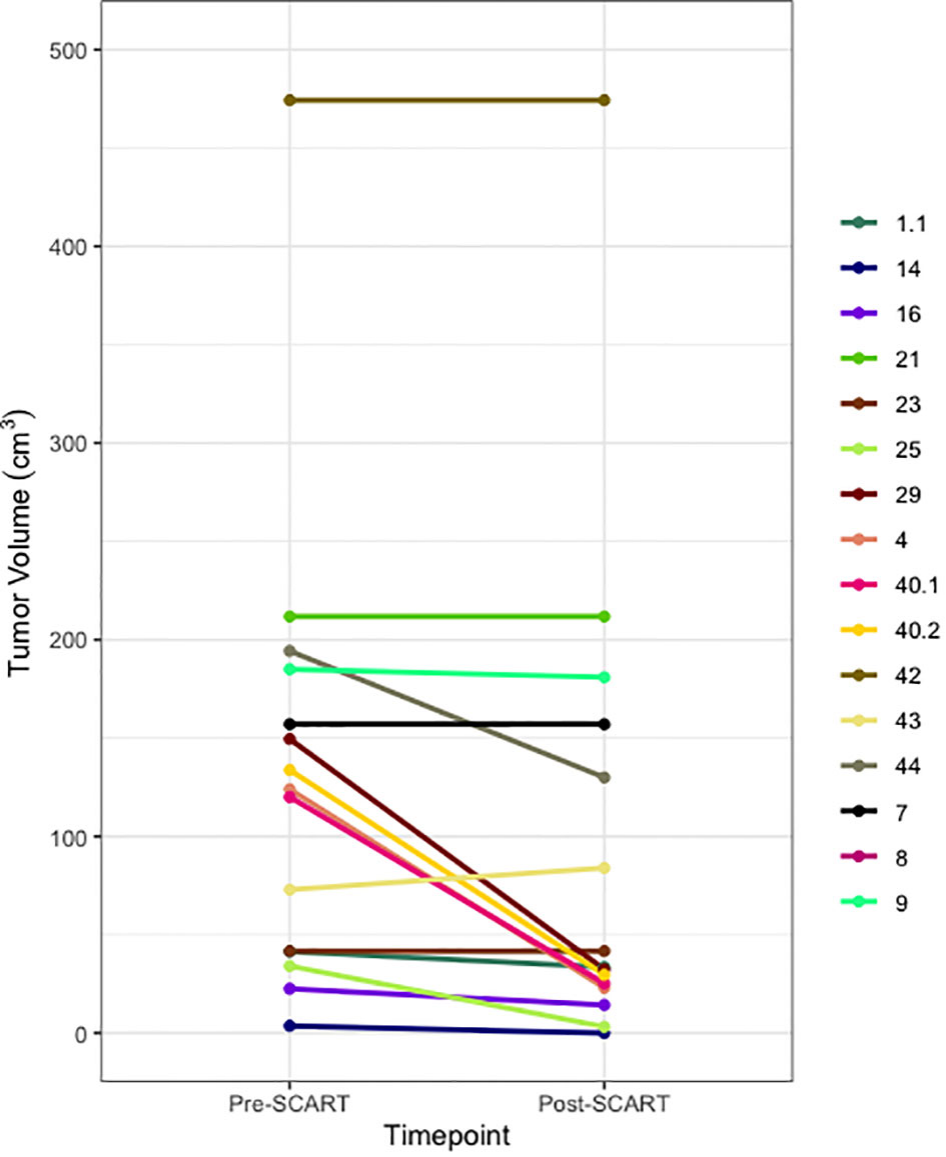
Spaghetti Plot for Changes in Tumor Size from Pre- to Post-SCART (N=16* tumors among 15 patients). *One patient’s tumor volume data (ID Name: JM) cannot be seen, as it was a large outlier and would not fit within the limits of the plot. Only 16 out of the 20 tumors among 15 patients are represented in this plot due to missing pre- and post-SCART data.

**Figure 3:**
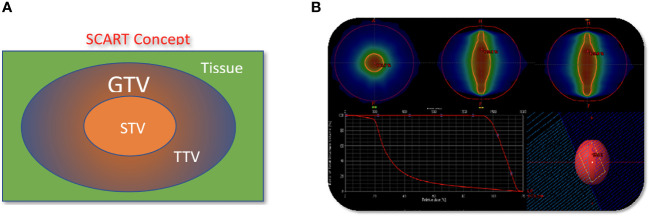
The concept and implementation of SCART. **(A)**, GTV (Gross Tumor Volume) in blue is the entire bulky tumor volume. STV (SCART- Treatment Volume) in orange is the central segment of GTV receiving ablative SCART dose. The cancer cells in STV tends to be hypoxic and cold in immune micro-environment. To kill these cells required ablative dose and the radiation ablation is likely to trigger the bystander effect and abscopal effect. TTV (Transitional Treatment Volume) is the rest of GTV volume outside of STV and partially/modestly irradiated and its heterogeneous dose transits from high, ablative SCART dose at STV border to a low, tissue-safe dose at GTV border. The cancer cells in TTV tend to be normoxic and warm in immune micro-environment. **(B)**: One or two co-planar 360 degree 6 MV photon arcs werewerearc was used with VMAT technology. The maximum field size was limited to SCART-PTV in the optimization, so that all the radiation fields from 360 degrees only irradiate and intersect at SCART-PTV, but not the whole GTV. This will generate the maximum dose gradient between SCART-PTV and GTV border. The SCART-PTV’s shape is typically a spindle shape and its dimension is proportional to the GTV dimension. The proportion is determined by the prescription dose (15~24Gy) and border dose (5Gy). The higher the prescription dose is, the smaller the proportion is. Many tests were done and the recommended proportion is listed in the following table.

Descriptive statistics were used to characterize demographic variables such as age and sex, as well as for tumor characteristics such as tumor site, tumor histology, tumor stage, and treated site. Patients’ treatment history, such as indicators for systemic therapy,Chemotherapy, and immunotherapy are described in the tables, along with indicators for some outcomes of interest (OS, PFS, LR) and the outcomes of interest themselves (OS, time to LR, TS) (**Tables 2**, **3**). Continuous variables such as age and gross tumor volume (GTV) were described using means and standard deviations. Categorical variables, including sex, tumor site, tumor stage, treated site, systemic therapy, chemotherapy, immunotherapy, and indicators for death, PFS, and LR were described using frequencies and percentages. Medians and ranges were utilized when describing skewed variables such as SCART dose, SCART dose at tumor edge, and outcome variables like OS and Time to LR. OS, PFS, and time to LR were estimated using Kaplan-Meier methodology, and distributions in the overall sample were visually displayed using Kaplan-Meier curves. A one sample t-test was used to compare tumor volumes pre- and post-SCART (**Figures 2**, **3**).

**Table 3:** Descriptive statistics for changes in tumor volume from pre to post SCART (N=16* tumors among 15 patients).

**Variable**	**Pre-SCART**		**Post-SCART**	**Change**	**p-value**
**Tumor Size** **(cm^3^), Median(range)**	183.10 (11.60,		37.64 (0.00,	-105.90 (-1,058.00,	<0.0001
	1,686.00)		628.00)	40.40)	

*Change was examined in only 16 of 20 tumors among 15 patients due to missing tumor volume data at either timepoint.

## Results

**Figure 4:**
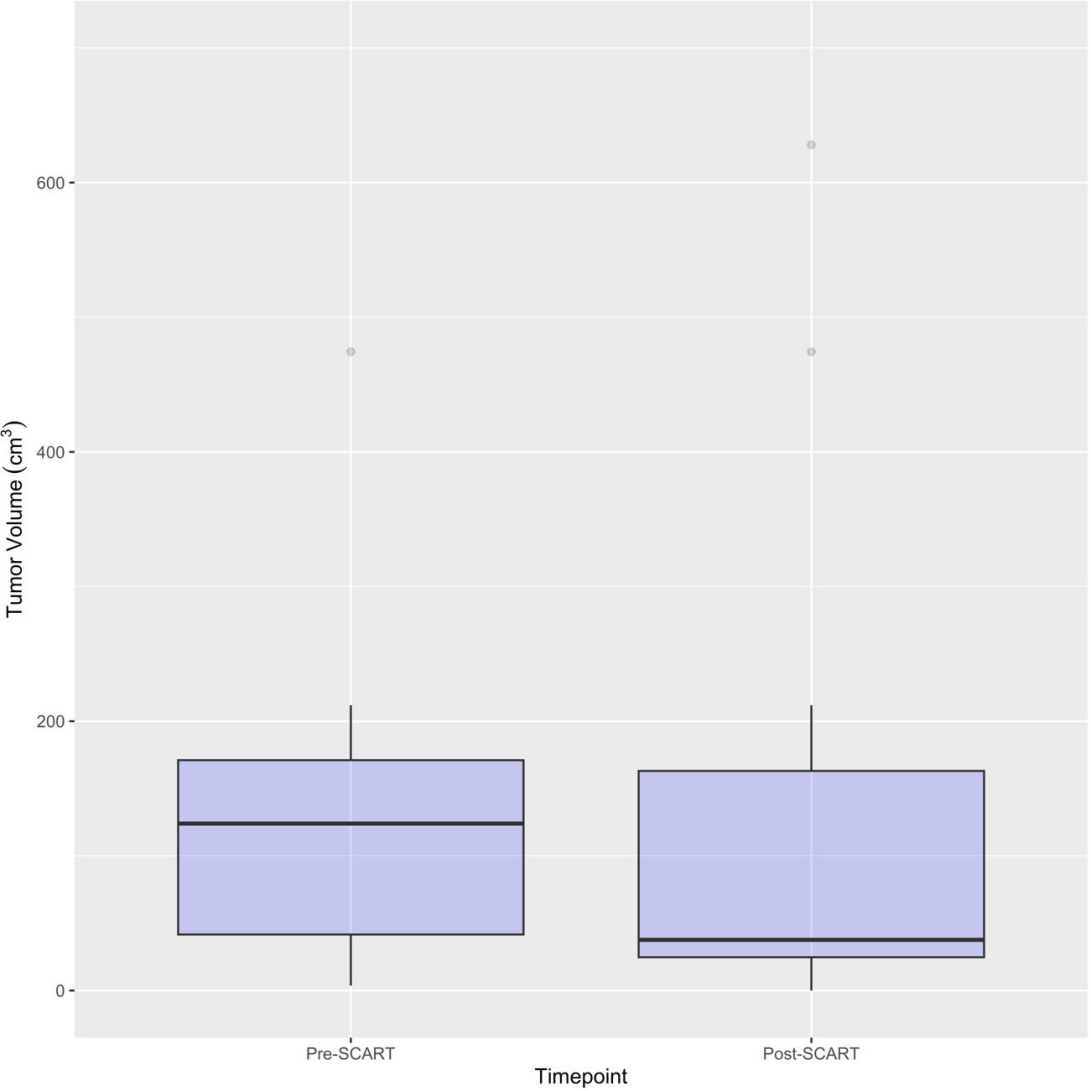
Boxplots for Pre- and Post-SCART Tumor Volumes (cm^3^) (N=16* tumors among 15 patients). *One patient’s tumor volume data (ID Name: JM) cannot be seen, as it was a large outlier and would not fit within the limits of the plot. Only 16 out of the 20 tumors among 15 patients are represented in this plot due to missing pre- and post-SCART data.

Descriptive statistics at the patient level (N= 17*) were used to characterize demographic variables such as age and sex, as well as some clinical and treatment characteristics such as systemic therapy, chemotherapy, immunotherapy, death, and overall survival (**Table 2A**). Descriptive statistics were also used to describe tumor characteristics like tumor site, tumor histology, tumor stage, treated site, GTV volume (cm^3^), SCART Dose (Gy), SCART dose at tumor edge (%), and local recurrence at the tumor level (N= 20 tumors among 17 patients). **Table 2B**: Continuous variables normally distributed, such as age, were described using means and standard deviations. Categorical variables, including sex, tumor site, tumor stage, histology, treated site, systemic therapy, chemotherapy, immunotherapy, and indicators for death and LR were described using frequencies and percents. Medians and ranges were utilized when describing non-normally distributed variables such as SCART dose and SCART dose at tumor edge, along with OS. OS was estimated using Kaplan-Meier methodology, and the distribution was visually displayed using a Kaplan-Meier curve (**Figure 4**). LR was described using descriptive statistics and not estimated using Kaplan-Meier methodology, as there was only one local recurrence in the entire dataset. A box plot (**Figure 2**) and spaghetti plot (**Figure 3**) were used to compare tumor volumes pre- and post-SCART. Descriptive statistics such as medians and ranges were used to describe the change in tumor volume from pre-SCART to post-SCART (**Table 3**) and to describe toxicities at the patient level (**Table 4A**) and at the toxicity level (**Table 4B**).

**Table 4A:** Descriptive statistics of toxicities at the patient level (N=17 patients).

Patient Level Data	(N=17 patients)
Number of toxicities	
** Mean (SD)**	1.12 (0.781)
** Median (Q1, Q3)**	1.00 (1.00, 1.00)
** Min, Max**	(0.00, 3.00)
Number of toxicities, N (%)	
** 0**	3 (17.6)
** 1**	10 (58.8)
** 2**	3 (17.6)
** 3**	1 (5.9)
**At least one toxicity = Yes, N (%)**	14 (82.4)

**Table 4B:** Descriptive statistics of toxicities at the tumor level (N=19* tumors among 14 patients).

Toxicity Level Data	(N=19* tumors)
Toxicity Area, N (%)	
** Cough**	1 (5.3)
** Fatigue**	4 (21.1)
** GI**	10 (52.6)
** Liver**	1 (5.3)
** Lung**	2 (10.5)
** Skin**	1 (5.3)
**Tumor Grade = 1, N (%)**	19 (100.0)

*19 toxicities among 14 patients with at least one toxicity. Two patients were removed due to missing data.

*One patient was removed from the data due to missing follow-up information.

## Results

### Sample characteristics

As can be seen in **Table 2A**, the average age of patients in the sample was 66 years (mean: 65.65, standard deviation: 17.39) and 10 patients or 58.8% of the sample were male. Exactly half of the sample had a tumor either in the liver (25.0%) or lung (25.0%). Almost a quarter (23.5%) of the sample had adenocarcinoma and most tumors (70.0%) of the sample had a stage IV tumor. As seen in **Table 2B**, 50% of tumors received a SCART dose of 15 Gy, and 75% of tumors received 30% of their total SCART dose at the tumor edge. The median decrease in tumor size was 105.90 cm^3^ (**Table 3**). The median time from the patient’s first SCART date to final SCART date was 2.71 months, and the range was 0.00 to 22.45 months. Based on the data, specifically the p-value (<0.0001), there is evidence that there is a statistically significant difference in tumor sizes between first visit volumes and post-SCART volumes, however this should be taken with caution considering the small sample size (N= 20tumors). The mean number of toxicities among 17 patients was 1.12 (SD: 0.781) (**Table 4A**). The median number of toxicities was one, and the minimum and maximum were 0 and 3. Out of those 17 patients, 14 had at least one toxicity (**Table 4A**). There were 19 toxicities examined in **Table 4B** among the 14 patients with at least one toxicity. Over half (52.6%) of the toxicities occurred in the GI area (**Table 4B**). All 19 toxicities were Grade 1 (**Table 4B**).

**Figure 4** visualizes overall survival among 17 patients with available data. Of these 17 patients, 8 died (47.1%). The percent of patients free from death at one year after the beginning of SCART treatment was 61.71. The median time to death was 12.50 months (**Figure 1**).

**Figures 2** and **3** provide visuals to compare tumor sizes pre- and post-SCART treatments. Out of the overall sample of 20 tumors, only 16 tumors had available data for both pre-SCART tumor volume and post-SCART tumor volume. However, one tumor (observation number 8) was an outlier and was not represented in these figures because they were beyond the limits of the y-axis. As seen in both figures, there appears to be a trend of tumor sizes decreasing from the patients’ first visit date, or pre-SCART, to their final volume post-SCART.

Among the cohort, four patients exhibited tumor volume reduction of less than 25%. Tragically, two patients succumbed within a two-month timeframe following SCART administration, precluding comprehensive monitoring of real-time progress. The remaining two patients were diagnosed with malignant mesenchymoma and mesothelioma, both recognized for their poor immunogenicity. It is postulated that these tumor types may exhibit limited responsiveness to SCART, possibly attributable to their diminished capacity to evoke a local immune response.

## Discussion

The radiation modality known as Spatially Fractionated Radiation Therapy (SFRT) entails the delivery of ablative and low-dose radiation to distinct regions within the same tumor. This approach may induce differing biological effects when contrasted with uniform irradiation across the entire tumor. In traditional radiation therapy, the sequential administration of a singular high ablative dose followed by fractionated lower doses has been observed to alter the tumor microenvironment from immunosuppressive to immunogenic. This alteration is characterized by increased infiltration of immune effector cells and a reduction in regulatory T cells (22–24).

Notably, clinical investigations have demonstrated instances of abscopal effects in patients receiving partial irradiation of bulky tumors. SBRT-PATHY, a fractionated radiation therapy designed for unresectable bulky tumors, capitalizes on radiation-hypoxia-induced non-targeted effects such as bystander and abscopal effects (20). Clinical trials employing “metabolism-guided” lattice radiotherapy, which utilizes radiation doses conducive to bystander, abscopal, and immunological effects, have reported a notable clinical response rate, including complete remission in a subset of stage IV bulky tumor patients (25). Preclinical research suggests that these bystander effects are mediated by cytokines (26), notably Tumor Necrosis Factor-Related Apoptosis-Inducing Ligand (TRAIL) (27) and Tumor Necrosis Factor (TNF) (28). The aforementioned studies underscore the potential of spatially fractionated radiation to enhance systemic or local responses, thereby indicating its promise as a therapeutic approach.

**Figure 5:**
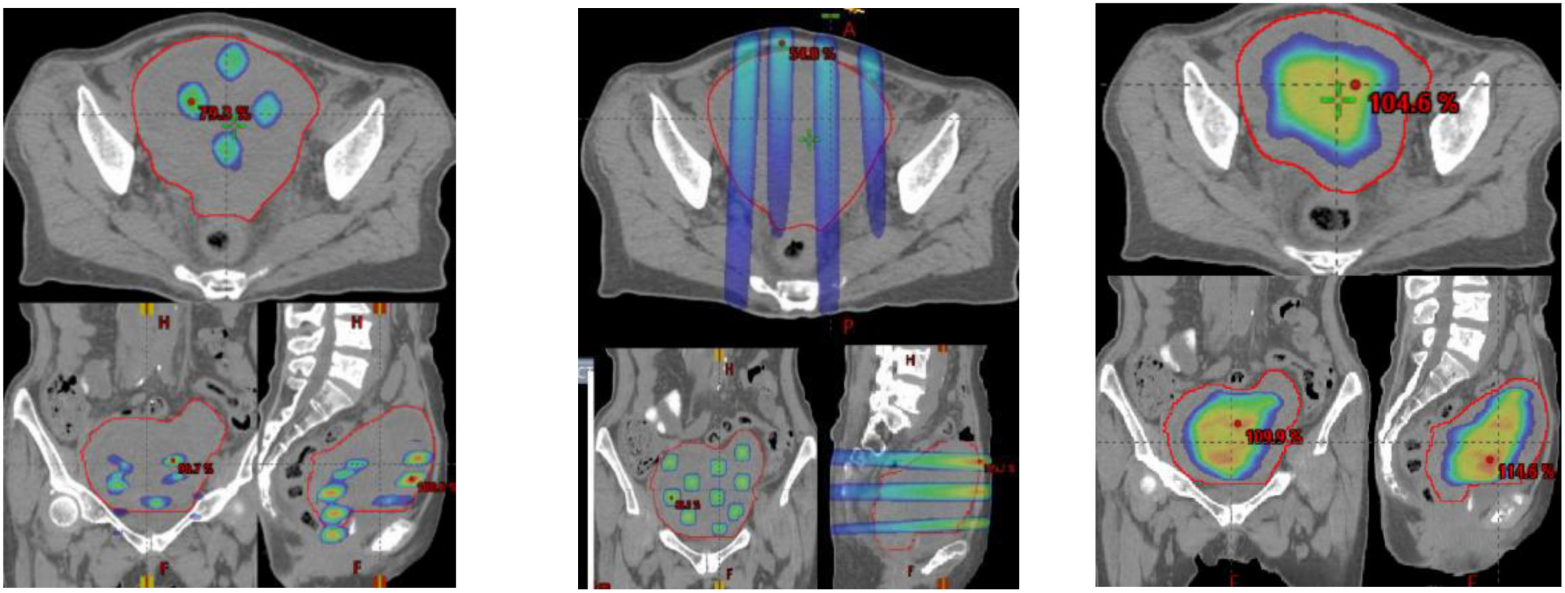
A clinical example of three SFRT methods. (GRID left, Lattice Middle, SCART Right) on a 672 cc Endometrial sarcoma with 15Gyx3fx as ablation prescription dose. The parameters are listed below, where the volume of tumor receiving the ablative prescription dose in SCART (67.6cc) is a or two folders larger than that of Grid (2.26 cc) and lattice (0.12 cc).

**Figure 6:**
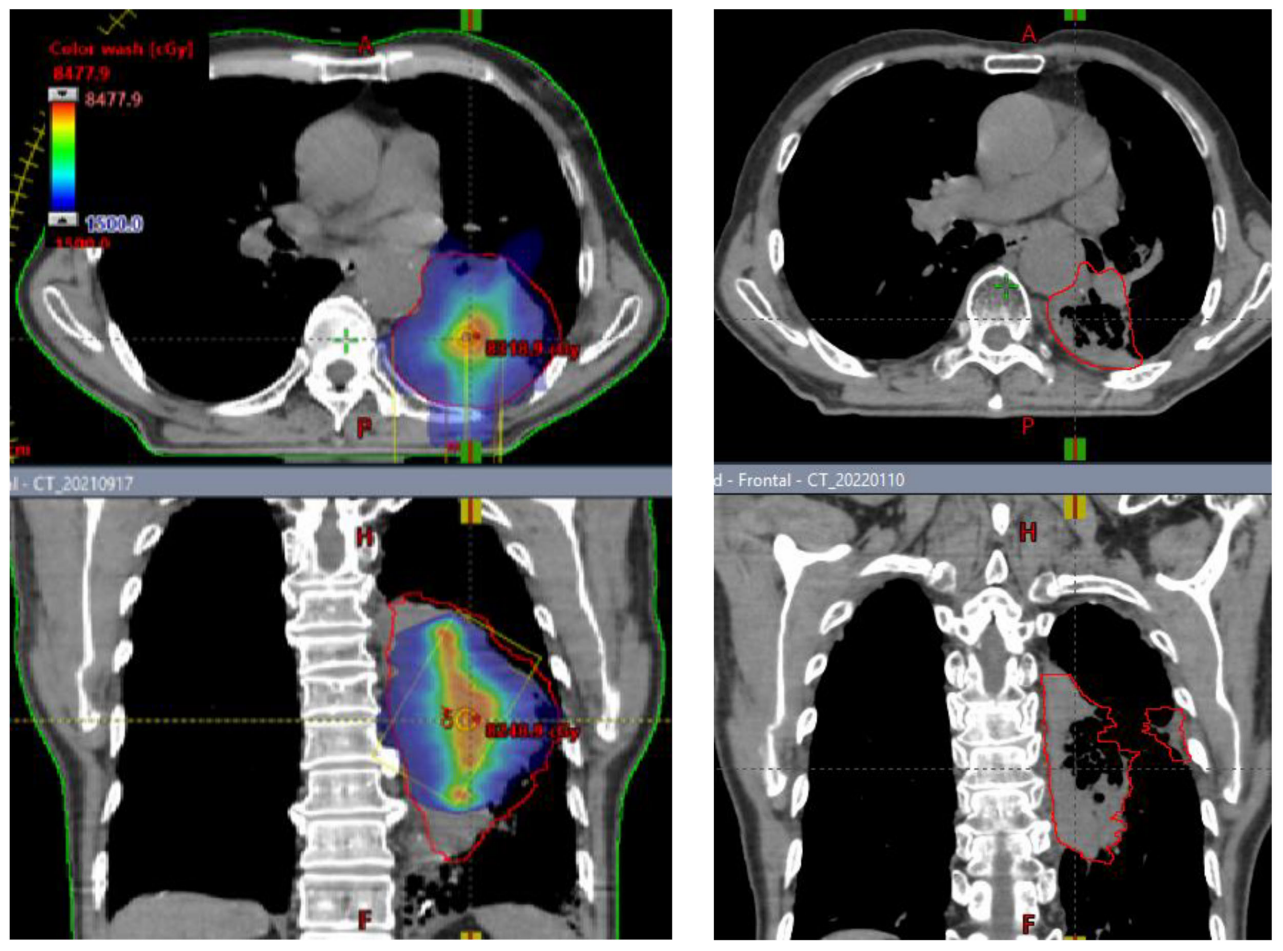
Representative Case: pre and post-SCART imaging for unresectable NSCLC.

SCART represents a method within the framework of Spatially Fractionated Radiation Therapy (SFRT) for manipulating the dose, fractionation, and area of ablative radiation within a tumor. A distinguishing feature of SCART, compared to approaches like GRID or LATTICE therapy, lies in its dose distribution (**Figure 5**). SCART can generate a much bigger high dose area intra-tumorally than GRID/Lattice. Notably, the dose delivered to the tumor periphery is considerably lower with SCART and can be systematically adjusted. We have successfully administered Stereotactic Body Radiation Therapy (SBRT) doses of up to 24 Gy x 3 to the central region of a tumor while maintaining a peripheral dose of 5 Gy. This approach achieves an ablative effect in a manner that ensures safety, particularly when the tumor is in close proximity to critical structures such as the brainstem, small bowel, or central bronchial tree (**Figure 6**).

Given SBRT can improve PFS and OS outcome with proper local control, SCART would be expected to show comparable results (29).

Multiple courses of Selective Conformal Ablative Radiation Therapy (SCART) can be administered to patients. Those who have previously undergone radiation therapy and subsequently experienced late radiation toxicity may encounter significant adverse effects on their quality of life and physical functioning. Options for re-irradiation in such cases are often limited. In the context of metastatic cancer, systemic treatment is typically the primary approach, with radiation therapy commonly offered for palliative purposes. There is a prevailing assumption that the presence of metastatic cancer implies widespread metastases, leading to the belief that localized treatment of individual metastatic tumors is futile and merely exposes patients to unnecessary interventions. However, direct treatment of these oligometastatic tumors has the potential to either extend survival or enhance the quality of life for affected patients through decreasing tumor burden and modulating human local and systemic immunological responses (30–33).

## Conclusion

In this current investigation, the administration of Stereotactic Central/Core Ablative Radiation Therapy (SCART) for recurrent or metastatic bulky tumors demonstrated favorable tolerability and safety, allowing for dose escalation up to the Maximum Tolerated Dose (MTD) of 24 Gy delivered in 3 fractions. Subsequent research endeavors involving a larger patient cohort are warranted to optimize the therapeutic efficacy of this highly promising, safe, and feasible treatment approach. SCART holds the potential to significantly augment our armamentarium in the ongoing struggle against cancer.

## Data availability statement

The original contributions presented in the study are included in the article/supplementary material. Further inquiries can be directed to the corresponding author.

## Ethics statement

The studies involving humans were approved by IRB of QuanZhou BinHai Hospital, Fujian, China. The studies were conducted in accordance with the local legislation and institutional requirements. The participants provided their written informed consent to participate in this study.

## Author contributions

JY: Writing – original draft, Writing – review & editing, Resources. WY: Writing – review & editing, Writing – original draft. QL: Writing – review & editing, Project administration. WQ: Writing – review & editing, Project administration, Investigation, Data curation. RK: Writing – review & editing, Software, Formal analysis. LW: Writing – review & editing, Investigation, Conceptualization. YL: Writing – review & editing, Investigation. SL: Writing – review & editing, Investigation. YL: Writing – original draft, Investigation, Formal analysis, Data curation. JL: Writing – original draft, Formal analysis, Data curation. WM: Writing – original draft. FM: Conceptualization, Methodology, Project administration, Writing – original draft, Writing – review & editing. TS: Writing – original draft, Supervision, Conceptualization. XW: Writing – review & editing, Supervision, Conceptualization. W-CY: Methodology, Resources, Writing – review & editing. EY: Investigation, Resources, Supervision, Writing – review & editing. AH: Writing – original draft, Methodology, Formal analysis, Data curation. AZ: Conceptualization, Methodology, Project administration, Writing – original draft, Writing – review & editing.
