# Supplementary material for: Stereotactic central/core ablative radiation therapy: results of a phase I study of a novel strategy to treat bulky tumor
Source: Front Oncol. 2024 May 24;14:1364627. doi: 10.3389/fonc.2024.1364627 (PMC11157688; doi:10.3389/fonc.2024.1364627)
Supplement: Supplementary file 1 [file Presentation_1.pdf]

Implementation of SCART

Stereotactic Core Ablative Radiation Therapy (SCART) can be highly effectively implemented using modern radiation machines such as the linear accelerator (Linac), robotic radiosurgery system (Cyberknife), or particle accelerator (Proton). Among these, Linac based Volumetric Modulated Arc Therapy (VMAT) is the most commonly employed in SCART. In VMAT, the Linac rotates around the patient, delivering continuous doses of radiation to the tumor from 360 degrees. This discussion primarily focuses on VMAT technology.

The typical prescription dose for SCART is 15~24Gy x3 fractions, with constraints on the surrounding tissue dose set at 3Gy or predominantly 5Gy per fraction. Patients undergo CT-based simulation using 2mm thin-slice CT for treatment planning. Contrast-enhanced CT, MRI, and PET/CT scans are routinely acquired and fused with the simulation CT to assist in tumor and organ at risk (OAR) delineation and contouring. The Gross Target Volume (GTV) is defined as the visibly bulky disease, while the Clinical Target Volume (CTV) coincides with the GTV and is not specifically highlighted in this study. The Stereotactic Treatment Volume (STV) is precisely defined as the region inside the GTV that receives the ablative dose during SCART treatment.

Design STV:

In the SCART planning, STV, but not the whole GTV nor TTV, is the true target of ablation and is pre-defined at the core of GTV prior to the planning optimization. In our design as Figure 1, the maximum opening of radiation arc is limited as the size of STV. These radiation fields only converge at STV, but not at TTV, and this arrangement will generate the highest possible dose gradient in TTV, the region between STV and GTV border.

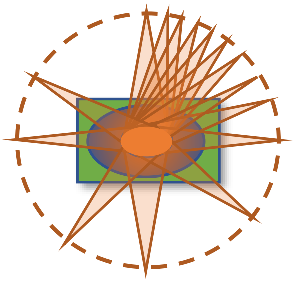

1.a

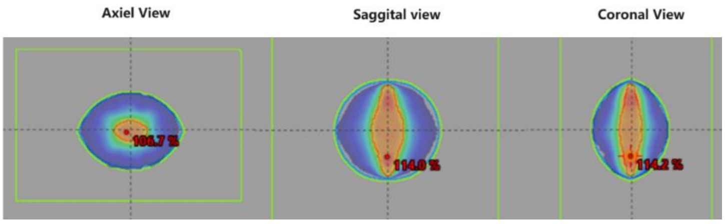

1.b

Figure 1 a. Illustration of VMAT arc irradiates the STV from 360 degrees. The maximum radiation field opening from various angels are limited to the projection of STV. These fields intersect at SSTV and the dose intensity falls off quickly at TTV, the surrounding region of GTV, to a modest dose level at the edge of GTV, which is safe to the surrounding tissue. 1.b Dose cloud of a hypothetical elliptic GTV and a elliptic SST in axial view, along with the spindle shape of SST in SI direction (Sagittal and Coronal view)

Using VMAT, the Stereotactic Treatment Volume (STV) is typically positioned at the center of the GTV, exhibiting a shape akin to the GTV in axial view and resembling a spindle in the superior-inferior (SI) view.

Defining Stereotactic Treatment Volume (STV) Dimensions in SCART

Determining the appropriate dimensions for the STV poses a challenge, as the dose gradient at the Transitional Treatment Volume (TTV) is intricately linked to the size of the STV. A delicate balance must be struck, considering that a larger STV results in a larger radiation field, slowing down the dose falloff in the surrounding area. Conversely, a larger STV leads to a smaller TTV, offering less space for the dose to attenuate. The goal is to identify the optimal STV dimensions, where the ablative prescription dose at the STV's edge precisely falls off to the tissue constraints dose at the GTV's edge. This ideal STV configuration is crucial for maximizing the elimination of hypoxic and radioresistant cancer cells in the tumor core and enhancing the likelihood of triggering a tumor-immune response.

Methodology for Dimension Determination: Using a SCART prescription dose of 15Gyx3 and a tissue constraints dose of 3Gyx3, hypothetical 10cm spherical tumors were employed for testing. The circular STV in axial view was incrementally increased by 1mm from 1cm, and each new STV was re-planned as the SCART target in VMAT treatment planning. The largest STV that

maintained sufficient prescription dose coverage without violating tissue dose constraints was identified as the optimal STV dimension, determined to be around 2.1 cm (21% of the sphere tumor's size in axial view).

STV/GTV Independence from GTV Dimension: Extending the tests to nine hypothetical spherical tumors ranging from 4cm to 20cm in diameter revealed a consistent proportionality between the dimension of the STV and GTV (approximately 20~22%, as detailed in the Table 1). This finding suggests that the STV dimension remains relatively independent of the GTV dimension across a range of tumor sizes, reinforcing the consistency of the proposed SCART methodology.

Table 1. The appropriate STV dimension and volume were listed in the nine hypothetical spherical tumors ranging from 4cm to 20cm in diameter. It is found that, regardless the tumor's dimension, there is a consistent proportionality between the dimension of the STV and GTV.

Tumor Diameter (cm)	Tumor Vol (cc)	STV Diameter (cm)	STV vol (cc)	$\frac{STVs\ Dimension}{GTV's\ Dimension}$	STV Vol %
4	32.8	0.9	0.9	**22.5%**	2.7%
6	112.1	1.2	2.8	**20.0%**	2.5%
8	267	1.8	8.3	**22.5%**	3.1%
10	522.6	2.1	15	**21.0%**	2.9%
12	904	2.5	22.5	**20.8%**	2.5%
14	1435	2.9	28.3	**20.7%**	2.0%
16	2143	3.4	47.5	**21.3%**	2.2%

18	3053	3.9	81.8	**21.7%**	2.7%
20	4189	4.2	127.5	**21.0%**	3.0%

STV/GTV depends on the Tissue constrain dose / SCART prescription dose

In our pursuit of refining Stereotactic Core Ablative Radiation Therapy (SCART), we explored the interplay between Stereotactic Treatment Volume (STV) and Gross Tumor Volume (GTV), specifically focusing on the relationship with Tissue Dose Constraints and SCART Prescription Dose.

To illuminate this intricate dynamic, we conducted targeted tests using a 10cm spherical tumor. Across four distinct prescription doses ranging from 15Gyx3 to 24Gyx3 and two tissue dose constraints (3Gyx3 or 5Gyx3), we observed a clear correlation listed in Table 2. The proportionality between STV and GTV appears to be significantly influenced by the prescription dose and tissue dose constraints. It is also found that STV/GTV proportionality is approximately same as Tissue Constrain Dose/SCART Prescription Dose proportionality.

Table 2: The list of STV and GTV proportion with the escalated prescription doses (15Gy ~ 24Gy) and two tissue dose constrains (3Gy and 5Gy) . It is found that STV/GTV dimension is similar to the Tissue Constrain Dose/SCART Prescription Dose.

Escalation level	prescription Dose	Tissue Dose Constrains	$\frac{Tissue\ Constrain}{Prescription\ Dose}$	$\frac{STV's\ Dimension}{GTV's\ Dimension}$	% of GTV Vol

**-1**	15 Gy x1	3 Gy x 1	**20%**	**21%**	3%
**-1**	15 Gy x1	5 Gy x 1	**33%**	**36%**	10.6%
**1**	15 Gy x2	5 Gy x 2	**33%**	**36%**	10.6%
**2**	15 Gy x3	5 Gy x 3	**33%**	**36%**	10.6%
**3**	18 Gy x3	5 Gy x 3	**28%**	**27%**	6.3%
**4**	21 Gy x3	5 Gy x 3	**24%**	**24%**	4.5%
**5**	24 Gy x3	5 Gy x 3	**21%**	**21%**	3.3%

As a rule of thumb for VMAT based SCART planning,

In Axial view

STV dimension = GTV dimension * Tissue Dose Constrains / Prescription Dose

In SI direction, STV is slight shorter of GTV and the STV typically looks like spindle in the sagittal and coronal view.

After STV being designed and cation will be added to review and edit it, if necessary, to avoid its overlapping with the serial organs, such as vessel or tracheal that resides inside of the tumor.

Optimized SCART Treatment Planning and Delivery

In the meticulous process of devising Stereotactic Core Ablative Radiation Therapy (SCART), we implemented a comprehensive treatment planning and beam arrangement strategy, ensuring precision and efficacy in every facet.

- **Beam Arrangement:**
  - One or two co-planar 360-degree 6 MV photon arcs were harnessed, employing Volumetric Modulated Arc Therapy (VMAT) technology.
  - As the exploration of non-coplanar beams enhances the dose gradient, we judiciously integrated non-coplanar beams or arcs whenever feasible.
  - Heterogeneity corrections applied in order to enhance dosimetry accuracy.
  - To refine optimization and minimize critical structure exposure, non-anatomical dose constraining ring structures were strategically incorporated.

Notably, intracranial targets, with their superior access to non-coplanar beams, resulted in a slightly larger/fatter STV in comparison to coplanar-only VMAT plans, attributing to its spindle-shaped nature.

- **Delivery:**
  - SCART sessions were scheduled every other day until the intended dose level was achieved or discontinued due to toxicity.

- Cone beam (CBCT) image guidance was meticulously employed in each treatment session, ensuring precision.

On average, 5302 monitor units (MU) were delivered in a streamlined 15-minute treatment time slot with average machine beam-on time of 8.9 min each fraction.
